# Differences in visual search behavior between expert and novice team sports athletes: A systematic review with meta-analysis

**DOI:** 10.3389/fpsyg.2022.1001066

**Published:** 2022-09-22

**Authors:** Ana Filipa Silva, José Afonso, António Sampaio, Nuno Pimenta, Ricardo Franco Lima, Henrique de Oliveira Castro, Rodrigo Ramirez-Campillo, Israel Teoldo, Hugo Sarmento, Francisco González Fernández, Agnieszka Kaczmarek, Anna Oniszczuk, Eugenia Murawska-Ciałowicz

**Affiliations:** ^1^Escola Superior Desporto e Lazer, Instituto Politécnico de Viana do Castelo, Viana do Castelo, Portugal; ^2^The Research Centre in Sports Sciences, Health Sciences and Human Development (CIDESD), Vila Real, Portugal; ^3^Sport Performance, Recreation, Innovation and Technology (SPRINT), Melgaço, Portugal; ^4^Faculty of Sport, Centre of Research, Education, Innovation, and Intervention in Sport (CIFI_2_D), University of Porto, Porto, Portugal; ^5^N2i, Polytechnic Institute of Maia, Maia, Portugal; ^6^Physical Education Department, Universidade Federal de Mato Grosso, Cuiabá, MT, Brazil; ^7^Department of Physical Activity Sciences, Universidad de Los Lagos, Santiago, Chile; ^8^Exercise and Rehabilitation Sciences Institute, School of Physical Therapy, Faculty of Rehabilitation Sciences, Universidad Andres Bello, Santiago, Chile; ^9^Centre of Research and Studies in Soccer (NUPEF), Universidade Federal de Viçosa, Viçosa, Brazil; ^10^University of Coimbra, Research Unit for Sport and Physical Activity (CIDAF), Faculty of Sport Sciences and Physical Education, Coimbra, Portugal; ^11^Department of Physical Education and Sport Sciences, University of Granada, Melilla, Spain; ^12^Department of Physiology and Biochemistry, University School of Physical Education, Wrocław, Poland; ^13^Wrocław University of Health and Sport Sciences, Wrocław, Poland

**Keywords:** decision-making, expertise, attention, gaze behavior, eye movements, motor behavior

## Abstract

**Background:**

For a long time, in sports, researchers have tried to understand an expert by comparing them with novices, raising the doubts if the visual search characteristics distinguish experts from novices. Therefore, the aim of the present study was to review and conduct a meta-analysis to evaluate the differences in visual search behavior between experts and novices in team sports athletes.

**Methods:**

This systematic review with meta-analysis followed the PRISMA 2020 and Cochrane's guidelines. Healthy team athletes were included, which engaged in regular practice, from any sex or competitive level, specifically classified *a priori* as expert or novice in the original research (i.e., if they were classified after the experiment, based on one of the tests, the study would be excluded). We considered only research published in peer-reviewed journals, with no limitations regarding date or language. It was considered healthy team sport athletes engaged in regular practice. The scenarios could be *in situ* or film-based. The databases of EBSCO (Academic Search Complete, Academic Search Ultimate, APA PsycArticles, and APA PsycINFO), PubMed, Scopus, SPORTDiscus, and Web of Science were used to perform the searches. The risk of bias was calculated through the RoBANS tool.

**Results:**

From a total of 6,257 records, of which 985 were duplicates, titles and abstracts of 5,272 were screened, and 45 required full-text analysis. Of those, 23 were excluded due to not fulfilling the eligibility criteria regarding participants. In the end, 22 studies were selected, however, as two studies were part of the same trial and were analyzed conjointly.

**Discussion:**

Experts showed to be older and with more years of practice. The ability to distinguish experts from novices was not so clear regarding the variables analyzed. This could be due to the strategies chosen in each study, which were specific to each scenario, and when grouping all together, it was lost information within non-representative averages. The distinction between experts and novices was not clear, showing a lot of heterogeneity in the included studies. The expert classification itself may have been the conditioning aspect for these results, retaining the doubt and the need for more studies in the field.

**Systematic review registration:**

The protocol was pre-registered in OSF (project https://osf.io/3j4qv/, register https://osf.io/dvk2n).

## Introduction

Decision-making, which is characterized as the players' ability to choose the most appropriate action from a vast number of possibilities to achieve a specific goal (Hastie, 2001), has been shown to play an important role in athletes to enable them to attain the highest performance in sports (Gréhaigne et al., 2001; Forsman et al., 2016). During a game, the athlete has to continuously analyze the environmental information and adjust their behavior by considering his/her teammate's and opponent's actions and contextual factors (Araujo and Davids, 2016). In fact, Newell (1986) noticed that the three interacting categories of constraints, organismic (i.e., performer), environmental, and task constraints, determine the optimal coordination and control of any activity.

The continuous perceptual-cognitive skill required for better decision-making refers to the ability to identify and attain environmental information, integrate with the existing knowledge, and select and execute an appropriate response (Marteniuk, 1976). That capacity relies strongly on visual search strategies, which differentiate experts from non-experts across a wide range of sports (Le Runigo et al., 2005). Indeed, knowing where and when to look is crucial for successful sports performance, since the visual display is immense and often saturated with information both relevant and irrelevant to the task (Mann et al., 2007). In fact, studies have shown that expert players (e.g., Vaeyens et al., 2007a; Roca et al., 2011; Ericsson et al., 2018), with higher skills in decision-making (Vickers, 1996b; Roca et al., 2012) and higher tactical behaviors (Williams and Davids, 1995; Cardoso et al., 2019), present superior ability to use perceptual-cognitive processes. Notably, more skilled players are able to adapt their visual search behaviors, according to the specificity of the situation (e.g., 1 vs. 1, 2 vs. 2, or 11 vs. 11), by utilizing more effective and assertive information search strategies (Vaeyens et al., 2007a). Besides, these players are able to better manage their cognitive effort when making decisions (Cardoso et al., 2019).

Studies showed that under certain conditions, a shift in the gaze is invariably preceded by a shift in attention (Shepherd et al., 1986; Kowler et al., 1995; Henderson, 2003). However, for many years, it was difficult to link attention with shifts in gaze (e.g., Posner, 1980). The literature now has strong evidence to confirm that when a saccade is made to a new location, there is a corresponding shift in attention in the direction of the saccade, meaning that when athletes shift their gaze to a new location, they also shift their attention to that location at least for a brief period (Vickers, 2009). This could be influenced by the visual field, i.e., normally what is measured is the central, or foveal vision, neglecting the peripheral vision. However, the central vision only represents 5 degrees of the visual field, but has the highest visual acuity (Millodot, 2017), allowing one to clearly see the visual stimuli. On the other hand, peripheral vision supports visual processing (Rosenholtz, 2016).

To analyze eye movement, the main variables analyzed are as follows: (i) fixations, (ii) saccades, and (iii) smooth pursuits (Bojko, 2013). When exploring fixations, studies have calculated their location (the relevant cues to which the subject is directing his/her gaze), their duration (the time spent on those cues), and the timing of fixations (the moment when the subject looks at those cues). There is a general acceptance that a fixation measures attention (Discombe and Cotterill, 2015). Saccades are characterized as a rapid movement from one fixation to another (Kowler, 2011). Those movements, which usually only take 30–80 ms, are useful for the brain to sample the visual environment (Discombe and Cotterill, 2015). Finally, smooth pursuits occur when we slowly track an object, but they are not voluntary movements (Kowler, 2011). Other less commonly recorded eye movements, which can only be detected by some high-end eye trackers, include microsaccades (actions to bring the drift back to the center of the fixation), tremors (very small eye movements during the fixation), drifts (automatically slow movements away from the center of the fixation), and glissades (a movement that the eye produces to correct for an overshot saccade) (Discombe and Cotterill, 2015). Another well-studied vision strategy variable in sport is the quiet eye, which represents the final fixation or tracking gaze made before the initiation of the action of importance in a motor coordination task (Vickers, 2007, 2009; Vickers et al., 2019). This occurs within 1° to 3° of visual angle (or less) for a minimum of 100 ms (Vickers, 2009; Dalton, 2021), allowing to perceive the task-relevant environmental cues and to master the motor plan for a successful upcoming task (Dalton, 2021).

The curiosity about what makes an expert athlete occupy a higher level when compared to other subjects started in the early 21st century (Dalton, 2021). In fact, the question begins by defining what really an expert athlete is, since the idea of the 10,000 h of deliberate practice of Ericsson (1996) has been shown to fail, persisting a lack of a clear definition of expert. In addition, a superior athletic performance could be readily apparent observed; however, perceptual-cognitive mechanisms that contribute to the experts' advantage are much less evident (Mann et al., 2007). Nevertheless, studies have been conducted comparing experts with non-expert athletes, mainly based on their performance level. In general, studies have shown that experts are better at detecting perceptual cues, make more efficient eye movements, and have better attentional processing compared with less accomplished athletes or nonathletes (Mann et al., 2007; Voss et al., 2010). This was reflected in a superior visual acuity (Laby et al., 1996; Uchida et al., 2013), better visual-perceptual and visual-cognitive abilities (Starkes and Ericsson, 2003; Williams et al., 2011), sensitivity (Hoffman et al., 1984), and better visual tracking abilities (Vickers and Adolphe, 1997) in expert athletes. In addition, elite athletes also exhibited fewer fixations of longer duration (Vaeyens et al., 2007b; Dalton, 2021) and spent more time fixating on key athletes and areas of space that could be exploited or exposed (Vaeyens et al., 2007b). The quiet eye has also been revealed to be longer by experts, and its onset is invariably earlier (Vickers, 2009). However, despite the fact that a considerable number of studies addressed visual assessment and training in athletes, relatively few have attempted to directly link these capabilities to on-field production statistics from competitive matches (Laby and Appelbaum, 2021). Moreover, it is hypothesized that the quality and accuracy of decisions can be influenced by different covariables, such as age, the relative age effect, or expertise (Sierra-Díaz et al., 2017; Araújo et al., 2019), as well as acute factors, such as fatigue (Russell et al., 2019).

The majority of the published work in this field has conducted laboratory-based simulations (e.g., Rivilla-García et al., 2013; Krzepota et al., 2016; Castro et al., 2017). However, this strategy could not be enough to fully understand the processes underlying decision-making (Dicks et al., 2010; Mann et al., 2010), since the perception-action couplings supporting decision-making are context-specific (Passos et al., 2008). Moreover, the small size of the scenario visualized (even large-screen projections are no match for the size of real-life events) and the analysis in 2D instead of 3D as in real life also limit the perception-action couplings. Therefore, evaluation in ecological contexts seems to play an important role to reveal the full nature of the expert advantage (Mann et al., 2010). Indeed, although greater performances are typically reported in the film-based simulations conducted, it could be possible that different processes may be used when viewing film simulations compared to those employed during the actual performance (Afonso et al., 2012).

To perform a controlled and precise movement, an accurate vision is essential (Roca et al., 2011; Williams et al., 2011; Laby and Appelbaum, 2021). In fact, these were the results of the meta-analysis of Mann et al. (2007); however, 15 more years passed, and more studies emerged. Hence, in a recent review (Laby and Appelbaum, 2021), it was highlighted that studies in this field offer promising but incomplete evidence that performance on visual assessments may correlate with game performance in competitive situations, not allowing to make strong conclusions. Therefore, the aim of the present study was to conduct a review with meta-analysis to reduce the doubts around this topic to seek a clear and stronger conclusion about the differences between experts and non-experts on visual search strategies in sports. Differences are expected to be found between experts and novices, with experts showing fewer fixations, but longer gaze durations and quiet eyes. This could also help to further understand the importance and how to implement new interventions to develop visual search in sports training.

## Methods

This systematic review with meta-analysis followed the PRISMA 2020 (Page et al., 2021) and Cochrane's guidelines (Higgins et al., 2019).

### Eligibility criteria

We considered only studies published in peer-reviewed journals, with no limitations regarding date or language. The eligibility criteria were set according to the PECOS (Participants, Exposure, Comparator, Outcome, Study design) framework (Morgan et al., 2018):

#### Participants

Healthy team sport athletes engaged in regular practice, from any sex, age, or competitive level, specifically classified *a priori* as expert or novice in the original research (i.e., if they were classified after the experiment, based on one of the tests, the study would be excluded). Alternative classifications were acceptable (e.g., more vs. less skilled, starters vs. non-starters, and more vs. less experienced), as long as the spirit of the comparison is preserved.

#### Exposure

Exposure to *in situ* or film-based match/game scenarios in which visual search behavior is assessed through eye-tracking technology (e.g., mobile, head-mounted eye trackers typically used in dynamic settings). Visual occlusion paradigms (i.e., where the timing of the occlusion is purposefully manipulated, instead of just having a final occlusion) and effects generated by implementing training programs and/or manipulating task constraints were not considered. In the same vein, studies where the participants had to engage in non-naturalistic actions (e.g., moving a joystick or pressing a button) were excluded.

#### Comparator

At least one group with an expert level was different from the main group (e.g., if one group is composed of experts, the other should be composed of novice athletes, and vice-versa).

#### Outcomes

The studies had to include at least one of the following outcomes: number of fixations, number of fixations per second, fixation location (i.e., areas of interest or interest areas), fixation duration, fixation duration per location, fixation order/sequence, visual field (i.e., area covered by the sum of central vision and peripheral vision), quiet eye duration, microsaccade and/or saccade amplitudes, durations, peak velocities, and accelerations.

#### Study design

Any study design, if at least one group of experts and one group of novices are included.

### Information sources

The databases of EBSCO (Academic Search Complete, Academic Search Ultimate, APA PsycArticles, and APA PsycINFO), PubMed, Scopus, SPORTDiscus, and Web of Science were used to perform the searches. On 1 October 2021, a data search was performed. No filters were used during the searches. After the automatic search, a manual search was conducted using the reference lists of the included articles. The final list was sent to two external experts (both with Ph.D. and publications related to the topic) for checking and identification of possible missing relevant articles. The experts suggested including three different studies; however, those papers were already been screened by authors and excluded as they did not fit the search requirements. Reviews about the topic were also consulted aiming to identify potential original articles that fit the scope; the general search strategy was complemented with “review” in the title, and searches were performed on PubMed and Google Scholar on 18 October 2021. Parallel to the list of included articles, an additional search for errata/corrections/corrigenda and retractions of the included studies was conducted (Higgins et al., 2019).

### Search strategy

Free text terms and Boolean operators (AND/OR) were applied to the title or abstract. No filters or limits were used. Some databases only perform wildcard searches (i.e., using the symbol ^*^) if words have a minimum of four letters, which was considered in our general search strategy:

Vision OR visual^*^ OR eye OR eyes OR gaze OR gazing OR ocular OR oculomotor OR decision^*^ OR anticipa^*^ OR “quiet eye” OR saccad^*^ OR “eye task” AND Sport^*^ OR athlet^*^ AND expert^*^ OR novice OR skill^*^ OR experience^*^

The fourth line of code was applied to full text/all text/any field (depending on the database):

“eye-track^*^” OR “eye track^*^” OR “fixation track^*^” OR “fixation-track^*^” OR “gaze-track^*^” OR “gaze track^*^” OR “eye movement”

Full search strategies and details for each database are presented in Table 1.

**Table 1:** Full search strategies for each database.

**Database**	**Specificities of the database**	**Search strategy**
EBSCO (Academic Search Ultimate, APA PsycArticles, and APA PsycInfo)	EBSCO does not allow combinations of title and abstract. To avoid multiple internal combinations (eight in total), we decided to use a more open search strategy in this database, with all code lines being open to “All text”.	(vision OR visual* OR eye OR eyes OR gaze OR gazing OR ocular OR oculomotor OR decision* OR anticipa* OR quiet eye OR saccad* OR eye task) AND (sport* OR athlete*) AND (expert* OR novice OR skill* OR experience*) AND (eye-track OR eye track OR fixation track* OR fixation-track* OR gaze-track* OR gaze track* OR eye movement)
PubMed	Nothing to report.	(((Vision[Title/Abstract] OR visual*[Title/Abstract] OR eye[Title/Abstract] OR eyes[Title/Abstract] OR gaze[Title/Abstract] OR gazing[Title/Abstract] OR ocular[Title/Abstract] OR oculomotor[Title/Abstract] OR decision*[Title/Abstract] OR anticipa*[Title/Abstract] OR “quiet eye”[Title/Abstract] OR saccad*[Title/Abstract] OR “eye task”[Title/Abstract]) AND (Sport*[Title/Abstract] OR athlet*[Title/Abstract])) AND (expert*[Title/Abstract] OR novice[Title/Abstract] OR skill*[Title/Abstract] OR experience*[Title/Abstract])) AND (“eye-track*” OR “eye track*” OR “fixation track*” OR “fixation-track*” OR “gaze-track*” OR “gaze track*” OR “eye movement”)
Scopus	In Scopus, the search for title or abstract also includes keywords.	(TITLE-ABS-KEY (vision OR visual* OR eye OR eyes OR gaze OR gazing OR ocular OR oculomotor OR decision* OR anticipa* OR “quiet eye” OR saccad* OR “eye task”) AND TITLE-ABS-KEY (sport* OR athlet*) AND TITLE-ABS-KEY (expert* OR novice OR skill* OR experience*) AND ALL (“eye-track*” OR “eye track*” OR “fixation track*” OR “fixation-track*” OR “gaze-track*” OR “gaze track*” OR “eye movement”))
SPORTDiscus	SPORTDiscus does not allow combinations of title and abstract. To avoid multiple internal combinations (eight in total), we decided to use a more open search strategy in this database, with all code lines being open to “All text”.	TX (Vision OR visual* OR eye OR eyes OR gaze OR gazing OR ocular OR oculomotor OR decision* OR anticipa* OR “quiet eye” OR saccad* OR “eye task”) AND TX (Sport* OR athlet*) AND TX (expert* OR novice OR skill* OR experience*) AND TX (“eye-track*” OR “eye track*” OR “fixation track*” OR “fixation-track*” OR “gaze-track*” OR “gaze track*” OR “eye movement”)
Web of Science	In Web of Science, the search for title or abstract also includes keywords, and is termed “topic”.	Query link: https://www.webofscience.com/wos/woscc/summary/ecaa9e68-ce0c-495e-9ab8-aae728eaa7bc-09cb81fe/relevance/1
		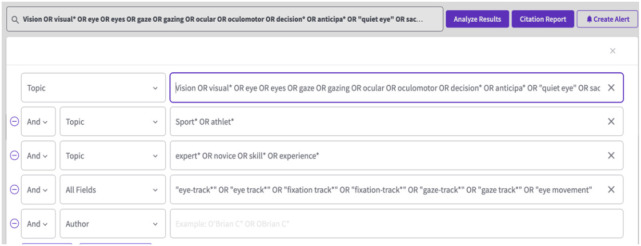

### Selection process

JA and HS independently screened each record. Disagreements were decided by IT. Automated removal of duplicates was performed using EndNote^TM^ 20.2 for Mac (Clarivate^TM^), but further manual removal of duplicates was required.

### Data collection process

RL and HC independently collected data. In case of disagreements, IT provided arbitrage. No automation tools were used.

### Data management

#### Data items

##### Primary outcomes

The primary outcomes were number of fixations, number of fixations per second, fixation location, fixation duration, fixation duration per location, visual field, quiet eye duration, microsaccade and/or saccade amplitudes, durations, peak velocities, and accelerations.

##### Secondary outcomes (when assessed)

The secondary outcomes were task reaction time, the efficacy of decision-making, and accuracy of motor responses.

Additional variables:

- Experiment-related variables: experimental setting (i.e., film-based, *in situ*, or both; in the case of film-based studies, report the area of the projection), description of exposure, eye-tracking specifications (model, sampling rate, gaze resolution, and noise), study's definition of fixation and/or saccades and microsaccades, and frequency of calibration (i.e., how many trials before re-calibration).
- Sample-related variables: specific sport, a competitive level (or equivalent concepts, such as training, experience, and/or skill level), age, sex, and years of practice.
- Other variables: study location (i.e., country), competing interests, and funding.

### Risk of bias assessment of studies

Since the studies were non-randomized by nature (i.e., experts were compared with novices) and the term exposure was more appropriate than intervention, Cochrane's RoBANS tool (Park et al., 2011) was used to assess the risk of bias arising from (i) selection of participants; (ii) confounding variables; (iii) measurement of exposure; (iv) blinding of outcome assessment; (v) incomplete outcome data; and (vi) selective outcome reporting. The risk of bias was considered similar for all primary outcomes, since data emerge from the same eye tracker device in each study. Therefore, only one risk of bias assessment was performed per study.

### Data synthesis

For continuous variables (e.g., fixation duration, fixation duration per location, visual field, quiet eye duration, microsaccade and saccade amplitudes, durations, peak velocities, and accelerations), studies were meta-analytically aggregated if three or more (Claudino et al., 2021) relatively homogeneous studies were available for the same outcome measure, with the main comparison being between experts and novices. A similar approach was planned for counts-related variables (e.g., number of fixations and number of fixations per second) by using statistical approaches previously proposed to combine dichotomous and continuous data (Higgins and Thomas, 2021). Effect sizes (ES; Hedge's g) were calculated using means and SDs from each dependent variable. For studies that reported standard errors, SDs were calculated by multiplying the standard error with the square root of the sample size (Higgins and Green, 2011; Lee et al., 2015). Depending on the outcome unit of measurement (e.g., degrees vs. radians) reported among included studies for meta-analysis, standard mean differences (SMDs) were also planned to be used.

The weight of trials was proportional to their individual standard errors through the application of an inverse variance random-effects model, as heterogeneity was expected (Deeks et al., 2008; Kontopantelis et al., 2013). The ES values were presented with 95% confidence intervals (95% CIs). The ES magnitudes were interpreted using the following scale: <0.2, trivial; 0.2–0.6, small; >0.6–1.2, moderate; >1.2–2.0, large; >2.0–4.0, very large; and >4.0, extremely large (Hopkins et al., 2009). The impact of study heterogeneity was assessed using the *I*^2^ statistic, with values of <25, 25–75%, and >75% representing low, moderate, and high levels, respectively (Higgins and Thompson, 2002).

Nominal variables (i.e., fixation location) were presented as original frequencies and percentages of the total. Locations were classified into seven categories, based on (Afonso et al., 2014; Roca et al., 2018) ball, opponent with the ball, opponent without the ball, teammate with the ball, teammate without the ball, functional space (i.e., visual pivots), and unclassified. Comparisons between experts and novices were performed using chi-square tests using the original frequencies (Schober and Vetter, 2019), with the effect size being calculated through Cramér (1946). Cramer's V is interpreted as a correlation: (McHugh, 2013), using arbitrary thresholds: very weak (0–0.19), weak (0.2–0.39), moderate (0.40–0.59), strong (0.6–0.79), and very strong (0.8–1) (The BMJ, 2021). For assessing the specific cells where differences emerged, adjusted standardized residuals were calculated, with |1.96| implying the cell had a number of cases significantly larger (or smaller, if negative) than expected (Agresti, 2002). Monte Carlo correction was used in cases where >20% of the cells had expected counts <5 (Irene et al., 2021).

Subgroup and/or sensitivity analyses were performed depending on the number of studies available in each comparison: (i) sex, (ii) sport, (iii) age groups, (iv) risk of bias, and (v) experimental setting (i.e., *in situ* vs. film-based). All analyses were carried out using the Comprehensive Meta-Analysis software (Version 2.0; Biostat, Englewood, NJ, USA) and IBM SPSS for Mac (Version 27; IBM, Armonk, NY, USA). The level of statistical significance was set at *p* < 0.05.

### Risk of reporting bias

The risk of reporting bias was explored for continuous variables (≥10 studies per outcome) (Sterne et al., 2011) using the Egger's test (Egger et al., 1997), with *p* < 0.05 implying the risk of bias. To adjust for the risk of reporting bias, a sensitivity analysis was conducted using the trim and fill method (Duval and Tweedie, 2000), with L_0_ as the default estimator for the number of missing studies (Shi and Lin, 2019). Computation of meta-regression was planned with at least 10 studies per covariate (Higgins et al., 2019).

## Results

### Study selection

Automated searches retrieved 6,257 records, of which 985 were duplicates. Titles and abstracts of 5,272 were screened, and 45 required full-text analysis. Of these, 23 were excluded due to not fulfilling eligibility criteria regarding participants (*n* = 6; Vaeyens et al., 2007a; Sáez-Gallego et al., 2013; Laffer et al., 2019; Cardoso et al., 2021a,b; Vítor de Assis et al., 2021), exposure (*n* = 6; Park, 2003; Jafarzadehpur et al., 2007; Núñez et al., 2009; Cañal-Bruland et al., 2011; Lex et al., 2015; Millard et al., 2020), comparators (*n* = 11; Shank and Haywood, 1987; Helsen and Starkes, 1999; Nagano et al., 2004; Zhang and Watanabe, 2005; North et al., 2009; Lee, 2010; van der Kamp, 2011; Roca et al., 2013; Wu et al., 2013; Uchida et al., 2014; Gorman et al., 2015), or outcomes (*n* = 1; Vater et al., 2019). Table 2 provides details about the 22 studies included in our review (Williams et al., 1994; Vickers, 1996b; Williams and Davids, 1997, 1998; Martell and Vickers, 2004; Vaeyens et al., 2007a; McRobert et al., 2009, 2011; Schorer and Baker, 2009; Roca et al., 2011; Afonso et al., 2012; Afonso and Mesquita, 2013; Rivilla-García et al., 2013; Vansteenkiste et al., 2014; Krzepota et al., 2016; Castro et al., 2017; Sarpeshkar et al., 2017; Klostermann et al., 2018; Abellán et al., 2019; Moeinirad et al., 2020; Natsuhara et al., 2020; Ribeiro et al., 2021). A manual search within the reference lists of these studies revealed 15 potentially relevant titles, of which 11 had appeared in our database searches. The abstracts of the remaining four studies were screened, with one study requiring full-text analysis and ending up fulfilling all eligibility criteria (Afonso and Mesquita, 2013). The search for reviews retrieved 10 relevant reviews on the topic, where 14 potentially relevant titles were identified. Of these, 10 titles had appeared in our initial searches, but four did not. Screening of their abstracts showed they did not fulfill all eligibility criteria. Two experts on the topic were consulted to ensure that all studies on the topic would be included. The suggestions made by experts were studies that had already passed through our analysis and had been excluded. No errata or retractions were found for any of the included studies.

**Table 2:** Study characteristics and main results.

**Study**	**Sample:** **1. Sex** **2. N** **3. Age**	**1. Years of practice and 2. Competitive level**	**1. Study location and** **2. Sport**	**1. Primary outcomes and** **2. Second outcomes**	**1. Experimental design and** **2. Experimental setting (film-based; in situation; both)**	**Exposure** **description**	**Eye tracker specifications**	**Study definition of fixation and/or saccades and microsaccades**	**Frequency of calibration**
Abellán et al. (2019)	1. Male 2. Expert (*n* = 10); High Intermediate (*n* = 11); Intermediate (*n* = 10) 3. Expert (18.2 ± 0.63); High Intermediate (16.45 ± 0.69); Intermediate (16.6 ± 0.84)	Expert (10.5); High Intermediate (7.27); Intermediate (7); Youth/Young athletes	1. Soccer field 2. Soccer	1. Visual behavior. 2. Hand-Eye coordination; Jump coordination; Hand movements, interception.	1. Between groups comparison 2. In situation	602 corner kicks were realized. In every corner kick, the goalkeeper tried to intercept the ball.	Applied Science Laboratories (ASL) 4000SU eye-movement	5 points fixated behind the goal.	The eye-movement recorder was calibrated using a 9-point reference grid so that the recorded indication of fixation position corresponded to the subject's visual gaze.
Afonso et al. (2012)	1. Female 2. Highly skilled (*n* = 15); Skilled (*n* = 12) 3. Highly skilled (19.1 ± 8.3); Skilled (17.3 ± 4.3)	1. Highly skilled (9.2 ± 6.5); Skilled (5.8 ± 2.3) 2. National level	1. Volleyball court 2. Volleyball	1. Visual behavior 2. Number of fixations; Number of locations; Verbal reports	1. Between groups comparison 2. In situation	Participants were instructed to take up their ready defensive position and to try to defend the ball. Participants were positioned in backcourt zone 6. Prior to engaging in the actual trials, the MobileEyeTM tracker was fitted to the participant's head and checked to ensure that it was comfortable and that interference with performance would be kept to a minimum. Participants stepped into the court and acted as backcourt defenders for as many trials as needed until six trials had been successfully ran.	Applied Science Laboratories (ASL) 3000 MobileEyeTM (30 Hz)	The eye movement registration system was calibrated using five non-linear points in the scene image so that the recorded indication of fixation position corresponded to each participant's point-of-gaze.	Re-calibration was conducted whenever: (a) the participant occasionally made a fall; (b) the ball was defended near to the face (implying a vigorous movement of the head); (c) the team performing the plays would commit to many fails, prolonging the duration of the testing; (d) the participant complained about sweating too much, with drops of sweat in the forehead or eyes' region, as such drops may impair the functioning of the infrared camera; and (e) the participants made arm movements that contacted the goggles and/or the cables. Additionally, random re-calibrations were at times conducted.
Afonso and Mesquita (2013)	1. Female 2. Skilled (*n* = 9); Less skilled (*n* = 6) 3. Skilled (16.1 ± 2.0); Less skilled (16.8 ± 2.0)	1. Not reported 2. Elite and less elite adults	1. Volleyball court 2. Volleyball	1. Decision-making 2. Number of fixation locations; mean number of fixations per trial; Mean fixation duration per trial; percentage of time spent in fixation on each area; Verbal report.	1. Between groups comparison 2. Film-based	Participants were presented with the six trials in the simulated task environment. These trials allowed participants to provide immediate retrospective verbal reports of thinking. Participants completed six trials and each individual test session was completed in ~20 min. The clips' order of presentation was kept consistent across all participants. Interviews were conducted after each trial and consisted in one question: “What were you thinking about while playing that point?”. In this protocol, immediately after each play the participant is removed from the court and inquired about his thoughts during the play. The participants had no time limit to respond.	Applied Science Laboratories (ASL) 3000 MobileEyeTM (30 Hz)	The eye-movement registration was calibrated using a 9-point grid so that the fixation mark corresponded precisely to the participant's point-of-gaze.	An eye calibration was performed for each participant to verify point-of-gaze before the trials and periodic calibration checks were conducted during testing.
Castro et al. (2017)	1. Male 2. Novices (*n* = 25) 3. U17: 16 ± 0.3; U18: 17.6 ± 0.9	1. U17: 3.2 ± 1.3; U18: 4 ± 0.9 2. Young/Youth	1. Sports Hall 2. Volleyball	1. Decision making 2. Number of visual fixations; Duration of visual fixation	1. Cohort Study 2. Film-based	Two scenes from each situation with durations of 4–6 s	Tracking SMI RED500®	Not reported	Not reported
Klostermann et al. (2018)	1. Male 2. Intermediate (*n* = 15); Highly skilled (*n* = 8) 3. Intermediate Skilled - 18.8 ± 0.6; Highly skilled - 17.7 ± 0.2	1. Intermediate skilled - 9.7 ± 5.6; Highly Skilled - 10.3 ± 3.7 2. Amateur; U19	1. Laboratory 2. Basketball	1. Visual behavior 2. Quiet eye behavior	1. Between groups comparison 2. In Situation	In the undefended game situation, after receiving a pass from another player located under the hoop, the player dribbled once and executed a jump shot from the free throw line at 4.25 m from the basketball hoop. In the defended game situation, the participants had to make jump shots from the free throw line as well, but the shooting attempts were made in 3 vs. 3 small-sided game situations with three attacking players (including the two participants) and three defensive players	Mobile Eye eye-tracking system (25 Hz, Applied Science Laboratories, Bedford, USA)	Mobile Eye systems were calibrated by fixating nine dots which were mounted to a white board.	Measurement accuracy of the Mobile Eye was verified after every tenth trial by adjusting the position of the fixation cursor, as necessary.
Krzepota et al. (2016)	1. Male 2. Experienced (*n* = 8); Less experience (*n* = 8); non-players (*n* = 8) 3. Experienced (22.2 ± 3.5); Less experience (23.5 ± 4.1); non-players (23.2 ± 4.0)	1. Experienced (12.4 ± 1.5); Less experience (11.2 ± 3.2) 2. Professional; University	1. Laboratory 2. Soccer	1. Visual behavior. 2. Number of fixations, Fixation duration; distribution of fixations, fixations across specific regions.	1. Between groups comparison. 2. Film-based.	Participants stood 4 m from a large screen (3.5 .5 m) on which the dribbling sequences were projected using a BENQ PU 9730 projector located behind and above. The recorded gaze activity clips, each ~5 s in duration, were collected for further analysis. The whole testing procedure, including instruction, calibration, and watching 20 offensive sequences, took about 4 min per each examined person.	Eye Tracking System mobile binocular (software at 60 Hz).	Seven regions of fixation were identified on the display to categorize the gaze position.	The appropriateness of the calibration procedure was verified prior to each trial to provide adequate accuracy of the system.
Martell and Vickers (2004)	1. Female 2. Elite (*n* = 6); U-22 (*n* = 6) 3. Elite (28 ± 4.73); Near Elite (21.67 ± 3.5)	1. Elite (11.16 ± 5.88); Near Elite (5.50 ± 2.35) 2. Elite and near elite	1. Ice Hockey Field 2. Ice Hockey	1. Visual behavior. 2. Gaze behaviors; type of gaze	1. Between groups comparison 2. In Situation	The participants skated a number of practice trials without opposition in order to become comfortable with the eye tracker and cable holder, who was an elite male player who shadowed Dp's movements. The conditions were counterbalanced in order to prevent guessing. A maximum of 24 trials were skated (12 in each condition) which was within the physical capabilities of the skaters. Total testing time took about 60 min.	Applied Sciences 501 mobile tracker	A saccade was coded when a rapid shift in gaze occurred between locations, with a minimum duration of 66.66 ms or two frames of video.	Before and after each trial, calibration was maintained.
McRobert et al. (2009) - same trial	1. Male 2. Skilled (*n* = 10); less skilled (*n* = 10) 3. Skilled (25.2 ± 6.8); less skilled (23.7 ± 4.1)	1. Skilled (13.7 ± 4.8); less skilled (11.2 ± 3.3) 2. Professional; Amateur	1. Laboratory 2. Cricket	1. Visual behavior; Anticipation task. 2. Visual search; Verbal report.	1. Between groups comparison 2. Both	Instructed to take up their normal batting stance holding a cricket bat and to play a stroke that would intercept the ball's anticipated flight path based online and length of the delivery observed. After playing the stroke, participants marked in pen the anticipated location of the ball when it passed the strike zone on a paper response sheet that depicted a scaled representation of the view from behind the stumps. Additional feedback on giving verbal reports was given when necessary.	Applied Science Laboratories 5001	The ASL eye-movement registration was calibrated using a 9-point grid.	Periodic calibration checks were conducted before and during
						testing. Participants then viewed all 36 video stimuli in the STE in randomized order. They were instructed to record the ball location on the response sheet after each trial and give retrospective verbal reports on every third trial and eight additional trials selected at random. The practice and test trials took ~90 min in total.			
McRobert et al. (2011) - same trial	1. Male 2. Skilled (*n* = 10); less skilled (*n* = 10) 3. Skilled (25.2 ± 6.8); less skilled (23.7 ± 4.1)	1. Skilled (13.7 ± 4.8); less skilled (11.2 ± 3.3) 2. Professional; Amateur	1. Laboratory 2. Cricket	1. Visual behavior. 2. Visual search; Fixation duration; Search rate; Verbal report.	1. Between groups comparison 2. Both	Participants took up their normal batting stance and were instructed to view and respond to the test film stimuli just as they would when facing a bowler in a real cricket match, including playing a batting stroke in response to each trial observed. On completion of each trial, participants were asked to mark a cross onto a paper response sheet, drawn to scale (i.e., 1:8 mm), that represented the x and y coordinates where the participant anticipated the ball to be when it passed the strike zone. Once the bowler initiated his run-up, participants were instructed to cease thinking out loud. Participants were given six practice trials from three fast bowlers not included in the experimental stimuli.	Applied Science Laboratories 5001	Were fitted with the eye tracker and calibrated to nine points on the calibration grid.	Not reported
Moeinirad et al. (2020)	1. Male 2. Skilled (*n* = 15); Near Expert (*n* = 12) 3. Skilled (9.33 ± 1.71); near expert (4.75 ± 1.2)	1. Skilled (23.13 ± 1.64); near expert (21.33 ± 4.0) 2. Professional; Semi-professional	1. Laboratory 2. Basketball	1. Visual behavior 2. Performance accuracy; Phase duration; Quiet eye duration	1. Between groups comparison 2. In situation	Participants took 10 shots in order to familiarize with the research environment, wore eye tracking glasses, and made five more attempts to get acquainted with the new situation. Then the eye tracking calibration was done using a three-dot method in which the dots were placed on a white screen. During the present study, the calibration was repeated after every 10 attempts. Each participant had to make at least 10 shots and miss at least 10 shots, although they were unaware of this process.	SensoMotoric Instruments Eye Tracking Glasses (SMI; Teltow, Germany; binocular) at a 60 Hz sample rate.	The eye tracking calibration was done using a three-dot method in which the dots were placed on a white screen.	Movements with the head to prevent the loss of the device's calibration.
Natsuhara et al. (2020)	1. Male 2. High level (*n* = 18); Middle level (*n* = 18)3. High level (19.7 ± 1.1); Middle level (20.1 ± 1.1)	1. High level (13.1 ± 1.7); Middle level (12.6 ± 1.8) 2. University Level; Amateur	1. Laboratory 2. Soccer	1. Decision-making 2. Visual search; Fixation duration; Verbal reports	1. Between Groups comparison 2. Film-based	15 different play videos were randomly presented twice, for a total of 30 times. However, participants were not told that the same videos were presented twice randomly. A ball was ejected according to the timing of the video presentation, and ejection was unified for each image. The ball was ejected 3 s after the presentation of the film, and the film was set to be occluded as soon as the ball arrived at the foot of the participant.	EMR-8b (eye movements were measured at a sampling rate of 30 Hz with the right eye monocular)	The study presented a near-life-size image that subtended a visual angle of around 72 in the horizontal direction and 55 in the vertical direction.	Authors calibrated the eye movement measurement system using a conventional 9-point reference grid according to the manufacturer's guidelines (recalibrated after 5 trials).
							The screen image subtended a visual angle of ~8, and the result of calculation by the trigonometric function showed an error level within 1 of the actual images.		
Ribeiro et al. (2021)	1. Female 2. U-17 (*n* = 6); U-14 (*n* = 6) 3. U-17 (15.83 ± 0.90); U-14 (13.29 ± 0.70 years)	1. U-14 (3.07 ± 0.48); U-17 (4.17 ± 1.11) 2. Youth	1. Laboratory 2. Handball	1. Visual behavior; Tactical knowledge 2. Visual fixation; Fixation duration	1. Between groups comparison 2. Film-based	15 scenes from videos of handball matches were used. Participants watched each scene and verbally declared (as quickly and accurately as possible) from the scene freezing, what would be the appropriate decision as if they were the player in possession of the ball and then justified this decision. Visual search data were collected while participants watched the scenes from handball matches that were displayed on the monitor of the equipment that registered the visual search.	Eye-Tracking SMI RED500	The eye tracker was calibrated by asking participants to fixate on targets presented on a screen across a nine-point grid.	Every time that participants sudden movements with the head to prevent the loss of the device's calibration.
Rivilla-García et al. (2013)	1. Male 2. Elite (*n* = 3), Amateur (*n* = 7) 3. 29.7 ± 5.4 years	1.14.7 ± 8.6 years 2. Elite and Amateur	1.7-m in front of a normal handball goal 2. Handball	1. Visual behavior. 2. Number of visual fixations	1. Between groups comparison 2. Film-based	Videos were shown of throws from 7 meters to different areas of the goal performed by players with different characteristics; The goalkeepers stood in front of the screen where the 14 throws from 7 meters were projected.	Tobii X120® Eye Tracker	Not reported	Not reported
Roca et al. (2011)	1. Male 2. Skilled (*n* = 10); less skilled (*n* = 10) 3. Skilled (23.6 ± 3.8); less skilled (24.3 ± 2.4)	1. Skilled (14.8 ± 3.3); less skilled (11.3 ± 4.1) 2. Professional; Semi-Professional	1. Laboratory 2. Soccer	1. Decision-making; Anticipation. 2. Visual search behaviors; Fixation duration; Fixation order; Verbal report	1. Experimental study 2. Both	Experiment 1 - At the end of each clip, participants were required to confirm “What the player in possession was going to do?” and “What decision the participant themselves made or were about to make at the moment of video occlusion¿‘ Participants completed 20 test trials and each individual test session was completed in ~45 min. The order of presentation of the clips was kept consistent across all participants. Experiment 2 - It was collected retrospective verbal reports directly after every trial (4 trials). Participants were tested individually in a quiet room, and each test session was completed in around 60 min.	Applied Science Laboratories	The system was calibrated using a reference of 6–9 nonlinear points on the scene image so that the recorded indication of fixation position corresponded to each participant's point-of-gaze.	Periodic calibration checks were conducted before and during presentation of the test film and minor adjustments made as necessary.
Sarpeshkar et al. (2017)	1. Male 2. Adult elite (*n* = 13); Youth elite (*n* = 10); Adult club (*n* = 10) 3. Adult elite (25.1); Youth elite (17.7); Adult club (31.7)	1. Not reported 2. Youth	1. Indoor facility 2. Cricket	1. Visual behavior. 2. Batting performance; gaze control	1. Experimental study 2. In situation	Participants faced 18 trials that followed a straight flightpath, and 4 were equally distributed across the three different ball-lengths and two lines (but were presented in the same randomized order for each participant). In the other block, participants faced a mixture of straight (random-straight) and swinging deliveries. This block consisted of 48 trials: 16 straight trials, 16 out-swing 7 trials, and 16 in-swing trials.	Mobile Eye monocular eye tracking system (25 Hz; Applied Science Laboratories, Bedford, MA)	The frequency of each of the three types of saccades was reported as the percentage of trials in which that type of saccade was performed, with the timing of each type of saccade reported relative to the moment of ball-release	Recalibration of the eye tracker was performed prior to, and after the completion of each 18 conditions, or if the unit was disturbed
Schorer and Baker (2009)	1. Male 2. Youngest group (*n* = 8); Youth (*n* = 5); Junior and adult (*n* = 9) 3. Youngest group: 14.4 ± 0.5; Youth: 16.8 ± 1.1; Junior: 19.2 ± 1.6; Adult: 27.3 ± 5.8; Senior:46.7 ± 3.8	1. Not reported 2. Young, youth, adult and senior	1.7-m in front of a normal handball goal 2. Handball	1. Decision making 2. Response execution; Response selection; Perceptual processes; General performance	1. Exploratory study 2. In situation	Participants were asked to conduct real goalkeeper movements at the beginning of a trial, they would stand in the middle of the goal and then react to the stimulus (e.g., reach to the lower right corner of the goal). These tasks were conducted in the order presented above, because the whole-body movements caused participants to sweat after a relatively short time and the eye-tracking system became less stable with sweat.	SMI iView X HED with a sample rate of 50 Hz.	The bicycle helmet was fitted to the participant's head and the eye-movement camera, and the scene camera were adjusted. The system was calibrated using a 5-point system.	Calibration was revaluated and adjusted, if necessary, prior to showing the second half of the scenes to the goalkeepers.
Vaeyens et al. (2007a)	1. Male 2. Elite group (*n* = 21); Sub-elite (*n* = 21); Regional (*n* = 23); students (22) 3. Elite group (14.7 ± 0.5); Sub-elite (14.6 ± 0.3); Regional (14.6 ± 0.6); students (14.5 ± 0.4)	1. Elite group (8.5 ± 1.4); Sub-elite (8.2 ± 1.1); Regional (7.3 ± 1.4); students (not reported) 2. Youth	1. Laboratory 2. Soccer	1. Decision-making 2. Reaction time; Decision time; Response accuracy; Search rate; Fixation location; Fixation order	1. Case control study 2. Film-based	Participants stood on two pressure sensitive switches and were required to make the correct tactical decision quickly and accurately when the ball was played in the direction of the player wearing the yellow vest. Thirty-three offensive patterns were selected for the experiment: two 2 vs. 1, ten 3 vs. 1, six 3 vs. 2, six 4 vs. 3, and nine 5 vs. 3 simulations. The order of presentation of film clips was randomized, with a comparable proportion of viewing conditions per block. All participants viewed clips in the same order. An intertrial interval of ~45 s was employed, and the entire test session was completed in around 45 min.	Applied Science Laboratories (ASL, Bedford, MA) software sampling at 60 Hz.;	Fixation locations were defined by comparing the point- of-gaze cursor, superimposed on the film sequence (i.e., the integrated eye–head data) with the coordinates obtained *via* the Eyenal program.	The calibration procedure was checked after the practice trials and between each of the three blocks of 11 test trials. Before each trial, an additional rapid calibration inspection was carried out to ensure system accuracy.
Vansteenkiste et al. (2014)	1. Female 2. Elite (*n* = 10); Intermediate (*n* = 10); Novice (*n* = 17) 3. Elite (20 ± 1.2); Intermediate (20.9 ± 1.8); Novice (20.1 ± 1.6)	1. Not reported 2. Professional; Amateur; Recreational	1. Laboratory 2. Volleyball	1. Visual behavior. 2. Reaction time; Response accuracy; Time course of gaze behavior	1. Between Groups comparison 2. Both	Each participant was then shown the 20 video clips of volleyball situations, which were randomized but in the same order for all participants and was asked to react as quickly and accurately as possible to the pass direction by moving in the same direction of the pass (imitating the movement of a counter). The participants were asked to look at the countdown which preceded each fragment so that the gaze direction was toward the center of the screen at the beginning of each trial.	Applied Science Laboratories Eye Tracking System, model 501; This system recorded the left eye movements at a frequency of 60 Hz with an infrared-sensitive camera using pupil position and corneal reflection.	Not reported	Not reported
Williams and Davids (1997) - same trial	1. Male 2. Experienced (*n* = 10); Less experience (*n* = 10) 3. Experienced - 20.8 ± 1.5; Less Experienced: 20.6 ± 2.1	1. Experienced – 12.4 ± 2.1; Less Experienced: 5.6 ± 2.5 2. Experienced: semi-professional; Less Experienced: University or Recreational	1. Laboratory 2. Soccer	1. Visual behavior; Selective attention. 2. Visual fixation; Fixation duration; Verbal report	1. Experimental study 2. In situation	The subjects were presented with three practice trials and 22 test trials.	Applied Science Laboratories (ASL) 4000SU	9-point reference grid so that the recorded indication of fixation position corresponded to the subject's visual gaze.	Rapid calibration check prior to each film trial.
Williams and Davids (1998)	1. Male 2. Experienced (*n* = 12); Less experience (*n* = 12) 3. Experienced - 24 ± 4.1; Less Experienced: 23 ± 4.0	1. Experienced – 13.4 ± 2.1; Less Experienced: 4.1 ± 2.5 2. Semi-professional	1. Laboratory 2. Soccer	1. Visual behavior; Selective attention 2. Movement time; Response time; Response accuracy	1. Experimental study 2. Both	Participants stood 5 m away from the screen so that the film image subtended a visual angle of ~40° in the horizontal and 35° in the vertical direction. Participants viewed each pattern of playas it developed and responded as quickly and accurately as possible by moving right, left, forward, or backward to simulate the interception of the pass. They were required to step on the same response pads as in the CRT experiment. Immediately following initiation of their response, the film was occluded to prevent participants gaining feedback on task performance.	Applied Science Laboratories 4000SU (at 50 Hz)	The eye movement recorder was calibrated using a nine-point reference grid.	Rapid calibration check prior to each trial
Williams et al. (1994) - same trial	1. Male 2. Experienced (*n* = 12); Less experience (*n* = 12) 3. Experienced - 24 ± 4.1; Less Experienced: 23 ± 4.0	1. Experienced – 13.4 ± 2.1; Less Experienced: 4.1 ± 2.5 2. Semi-professional	1. Laboratory 2. Soccer	1. Visual behavior. 2. Anticipation; Eye fixation; reaction time	1. Experimental study 2. Film-based	Participants were required to respond as quickly and as accurately as possible when stimulus was presented (black reference square that surrounded one of the 10 grid numbers). The test film included three practice trials and 22 test trials.	An Applied Science Laboratories (ASL; Waltham, MA) 4000 SU) at 50 Hz	The system measured pupil position as well as corneal reflex, with the relative position of these features being used to compute visual gaze with respect to the optics.	Rapid Calibration check prior to each film trial.
Vickers (1996a)	1. Female 2. Expert (*n* = 8); Near-Experts (*n* = 8) 3. Experts – 21.3 ± 2.5; Near-Experts: 20.8 ± 4.7	1. Experts – 10.1 ± 3.6; Near-Experts: 9.5 ± 3.1 2. Professional	1. Sports Hall 2. Basketball	1. Visual behavior. 2. Accuracy; Gaze behaviors; Fixation; Quiet eye	1. Cohort Study 2. In situation	Subjects took consecutive free throws until they had made 10 hits and 10 misses, a research goal of which they were unaware.	Applied Science Laboratories (ASL) - Panasonic Special Effects Generator, Model WJ 4600a.	The ASL system measures the positions of two features of the eye: the pupil and the corneal reflex positions of two features of the eye: the pupil and the corneal reflex (CR).	Recalibration was accomplished in a few seconds and performed an average of one to two times per subject, usually during the practice trials.

Importantly, the studies of McRobert et al. (2009, 2011) were part of the same trial, and so were analyzed conjointly. Likewise, the studies of Williams et al. (1994) and Williams and Davids (1997) were also part of the same trial, and were analyzed conjointly.

### Study characteristics

Table 2 summarizes all the characteristics of the studies included in this review. Most of the studies (~73%, corresponding to 16 studies) included only men (Williams et al., 1994; Williams and Davids, 1997, 1998; Vaeyens et al., 2007a; McRobert et al., 2009, 2011; Schorer and Baker, 2009; Roca et al., 2011; Rivilla-García et al., 2013; Krzepota et al., 2016; Castro et al., 2017; Sarpeshkar et al., 2017; Klostermann et al., 2018; Abellán et al., 2019; Moeinirad et al., 2020; Natsuhara et al., 2020). Conversely, the remaining studies only included women, as detailed in Table 2. Although the majority of the studies included experts or considered highly skilled athletes, six of them also analyzed youth athletes (Vaeyens et al., 2007a; Schorer and Baker, 2009; Castro et al., 2017; Sarpeshkar et al., 2017; Abellán et al., 2019; Ribeiro et al., 2021). The sports studies varied among soccer (*n* = 8), volleyball (*n* = 4), basketball (*n* = 3), cricket (*n* = 3), handball (*n* = 3), and ice hockey (*n* = 1); nevertheless, 13 studies conducted laboratory procedures. Considering the primary outcome, two main topics were analyzed: visual behavior (Williams et al., 1994; Vickers, 1996b; Williams and Davids, 1997, 1998; Martell and Vickers, 2004; McRobert et al., 2009, 2011; Afonso et al., 2012; Rivilla-García et al., 2013; Vansteenkiste et al., 2014; Krzepota et al., 2016; Sarpeshkar et al., 2017; Klostermann et al., 2018; Abellán et al., 2019; Moeinirad et al., 2020; Ribeiro et al., 2021) and the decision-making (Vaeyens et al., 2007a; Schorer and Baker, 2009; Roca et al., 2011; Afonso and Mesquita, 2013; Castro et al., 2017; Natsuhara et al., 2020). Considering the second outcome, the focus was mainly to characterize and compare between different levels of expertise, the number of fixations, and the number of locations, with some studies including verbal reports and the quiet eye analysis.

### Risk of bias in individual studies

The Risk of Bias Assessment Tool for Non-Randomized Studies (RoBANS) was used to assess the risk of bias in included studies. This tool contains guidelines for evaluation based on six categories, each to be assessed as “high risk”, unclear risk, or “low risk” (Kim et al., 2013). The RoBANS assessment was conducted by two authors independently (JA and AFS). Disagreements were resolved by consensus or consultation with a third assessor (IT) when required.

Considering the participants' selection, a low risk of bias was identified in 70% of the studies. The remaining 30% presented high risk because the expertise or skill level was pre-stipulated by experts, without an objective referral to the playing level. Likewise, a low risk of bias was reported in 70% of the studies when analyzing the issue of confounding variables, since most trials provided familiarization with the testing procedures. However, in some studies, there was no familiarization with testing procedures, which may have influenced the results, due to a learning effect. Although most studies had a low risk of bias for incomplete outcome data (75%), studies often only reported the final sample, and there was no clear indication of whether the included participants were part of a larger sample of the initially recruited group. Occasionally, only a small subset of visual search data would be selected for analysis. The major concerns with the risk of bias were related to the measurement (eye-tracking data) and the building outcome assessment, both presenting a high risk in 55% of the studies. In the first issue, it was observed that the digitalization of images was often performed by a single tester, unblinded to the skill level. However, often this was complemented by a second assessment, by independent raters. When this was not the case, the risk of bias in measurement became high. Regarding the blinding outcome assessment, it was registered that, usually, testers were not blinded, but in some studies, a second, independent tester provided inter-rater reliability calculations. In cases where this did not happen, we judged the study to be at high risk for blinding outcome assessment. Finally, considering the selective outcome reporting, no study had a pre-registered or pre-published protocol against which to compare the manuscript. Therefore, 90% of the studies were found to be unclear regarding whether the reporting outcome was complete or selective.

### Chronological age

**Figure 1:**
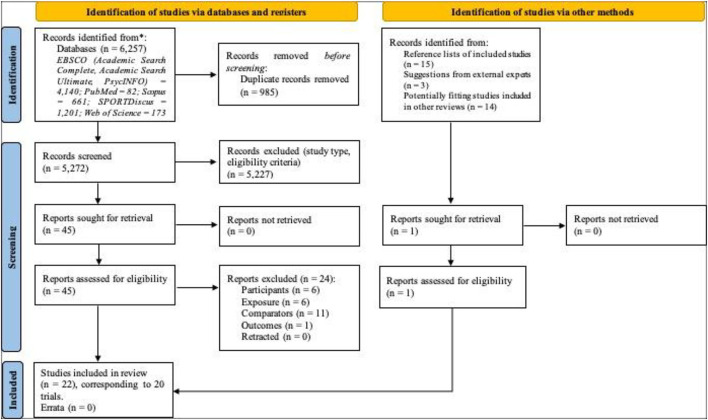
PRISMA 2020 flow diagram.

**Figure 2:**
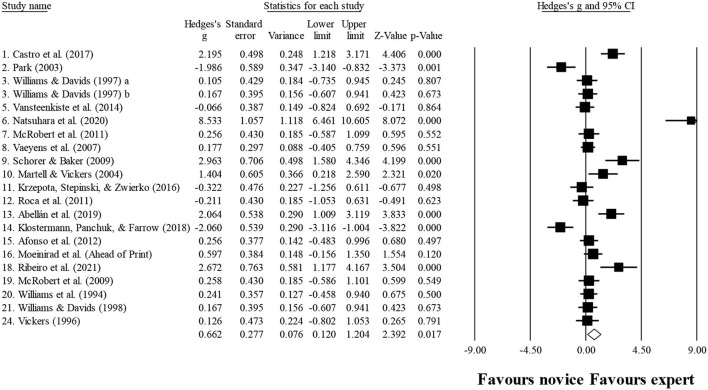
Chronological age: greater chronological age was noted for expert athletes compared to novice athletes. Black squares: individual studies. Its size represents their relative weights. White rhomboid: summary value.

Twenty studies provided data for chronological age, involving 20 expert and 20 novice groups (pooled *n* = 474). Results showed a moderate effect, with greater chronological age for expert athletes when compared to novice athletes (ES = 0.66; 95% CI = 0.12–1.20; *p* = 0.017; Figure 1; *I*^2^ = 86.9%; Egger test = 0.0139, with adjusted value at ES = 1.03; 95% CI = 0.43–1.64). When results were analyzed as per athletes' involvement in their respective sports (Figure 2), no significant moderator effect was noted for the type of sport (*p* = 0.290 between groups), involving basketball (three studies; *I*^2^ = 88.0%), soccer (nine studies; *I*^2^ = 89.3%), and volleyball (four studies; *I*^2^ = 90.1%) athletes. Other sports were not included in the moderator analysis, as less than three studies were available.

### Years of experience

**Figure 3:**
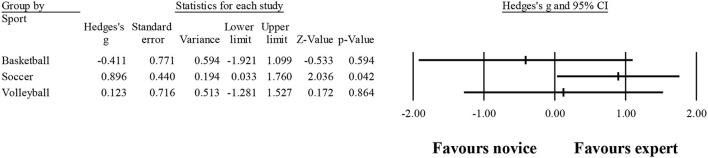
Chronological age moderated by type of sport: no significant moderator effect was noted for the type of sport (*p* = 0.290 between groups).

**Figure 4:**
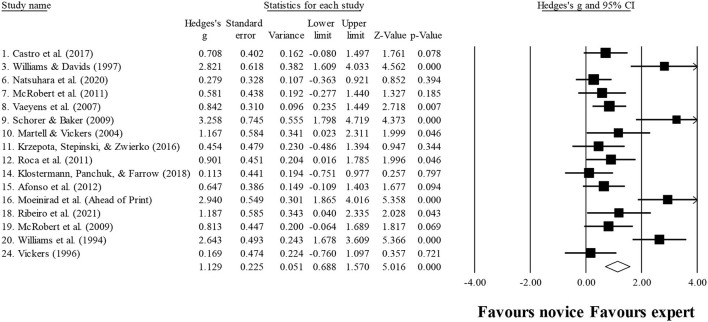
Years of experience: greater years of experience were noted for expert athletes compared to novice athletes. Black squares: individual studies. Its size represents their relative weights. White rhomboid: summary value.

Sixteen studies provided data for years of experience, involving 16 expert and 16 novice groups (pooled *n* = 363). Results showed a moderate effect, with greater years of experience for expert athletes when compared to novice athletes (ES = 1.13; 95% CI = 0.69–1.57; *p* < 0.001; Figure 3; *I*^2^ = 73.9%; Egger test = 0.007, with adjusted value at ES = 1.21, 95% CI = 0.76–1.65). When results were analyzed as per athletes' involvement in their respective sports (Figure 4), no significant moderator effect was noted for the type of sport (*p* = 0.835 between groups), involving basketball (three studies; *I*^2^ = 89.7%) and soccer (six studies; *I*^2^ = 81.0%) athletes. Other sports were not included in the moderator analysis, as less than three studies were available.

### Number of fixations

**Figure 5:**
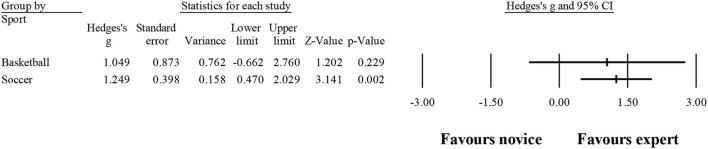
Years of experience moderated by type of sport: no significant moderator effect was noted for the type of sport (*p* = 0.835 between groups).

**Figure 6:**
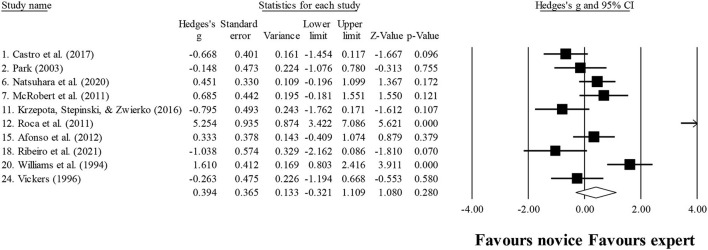
Number of fixations: greater (although not significant) number of fixations was noted for expert athletes compared to novice athletes. Black squares: individual studies. Its size represents their relative weights. White rhomboid: summary value.

Ten studies provided data for the number of fixations, involving 10 expert and 10 novice groups (pooled *n* = 218). Results showed a small effect, with greater (although not significant) number of fixations for expert athletes when compared to novice athletes (ES = 0.39; 95% CI = −0.32 to 1.11; *p* = 0.280; Figure 5; *I*^2^ = 84.4%; Egger test = 0.373). When results were analyzed as per athletes' involvement in their respective sports (Figure 6), a moderator effect (although not significant; *p* = 0.078 between groups) was noted for the type of sport, involving soccer (four studies; *I*^2^ = 92.1%) and volleyball (three studies; *I*^2^ = 39.4%) athletes. Other sports were not included in the moderator analysis, as less than three studies were available.

### Fixation duration

**Figure 7:**
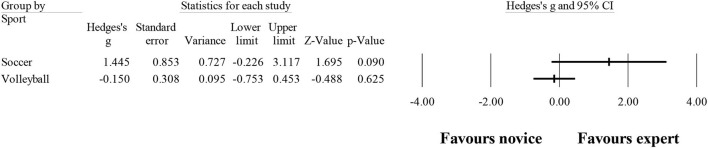
Number of fixations moderated by type of sport: no significant moderator effect was noted for the type of sport (*p* = 0.078 between groups).

**Figure 8:**
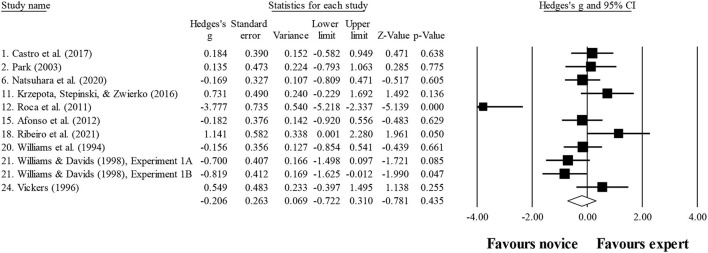
Fixation duration: similar fixation duration was noted for expert athletes compared to novice athletes. Black squares: individual studies. Its size represents their relative weights. White rhomboid: summary value.

Ten studies provided data for fixation duration, involving 11 expert and 11 novice groups (pooled *n* = 246). Results showed a small effect, with lower (although no significant) fixation duration for expert athletes when compared to novice athletes (ES = −0.21; 95% CI = −0.72 to 0.31; *p* = 0.435; Figure 7; *I*^2^ = 75.1%; Egger test = 0.509). When results were analyzed as per athletes' involvement in their respective sports (Figure 8), no moderator effect (*p* = 0.138 between groups) was noted for the type of sport, involving soccer (six studies; *I*^2^ = 82.7%) and volleyball (three studies; *I*^2^ = 0.0%) athletes. Other sports were not included in the moderator analysis, as less than three studies were available.

### Quiet eye duration

**Figure 9:**
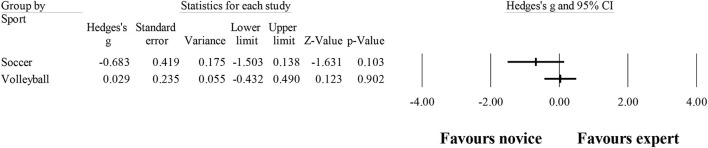
Fixation duration moderated by type of sport: no significant moderator effect was noted for type of sport (*p* = 0.138 between groups).

Three studies provided data for quiet eye duration, involving eight expert and eight novice groups (pooled *n* = 174). Results showed a small effect, with greater (although no significant) quiet eye duration for expert athletes when compared to novice athletes (ES = 0.34; 95% CI = −0.44 to 1.11; *p* = 0.396; Figure 9; *I*^2^ = 82.9%). Moderator analyses as per athletes' type of sport were precluded, as less than three studies were available.

### Other outcomes

The fixation duration per location, fixation order/sequence, visual field (i.e., area covered by the sum of central vision and peripheral vision), and microsaccade and/or saccade amplitudes, durations, peak velocities, and accelerations were not included in the present meta-analysis as initially planned, since not enough or no information was given in the included studies that would allow analysis and subsequent discussion and conclusion.

## Discussion

The present study aimed to review and conduct a meta-analysis to examine the differences between experts and non-experts in visual search. In contrast to what was hypothesized, results showed that the ability to distinguish experts from novices was not so clear regarding the variables analyzed. This could be due to the strategies chosen in each study, which were specific to each scenario, and when grouping all together, it was lost information within non-representative averages. Considering the participants included, not surprisingly, it was shown that experts were older and accumulated more years of practice. The analysis, by sport, revealed a moderate effect only on the number of fixations. Altogether, these results seem to highlight that chronological age and years of practice could really improve the athletes' level, and a visual search analysis should be conducted regarding the sport. Nevertheless, more studies need to be conducted in different sports to strengthen further conclusions.

A possible variable that could affect our results was the ambiguous definition of an expert athlete. In fact, in a review by Swann et al. (2015), at least eight different criteria were used to define an expert athlete. The most frequent criteria to distinguish an elite from a non-elite was if the athlete had an international and/or national competitive level. The athletes' experience occupied the second place (49% of the sample), and the professionalism characteristic took the third place, with almost 30% of the studies included. Clearly, such imprecision in the criteria used to define athletes as expert threatens the validity of research on expertise in sport. More recently, McKay et al. (2022) reinforce the importance of a clear definition of athlete's level, highlighting that athletic success might be explained by different attributes, with the athletic caliber influencing intervention results. Therefore, the authors presented a framework of five levels, with clear items to classify participants, which could help in future studies if we are really getting information from expert athletes. In fact, this could be the reason why, in the present study, the experts were shown to be older and with more years of practice than novices.

The number of fixations, which is characterized as the time spent looking at a particular location, provides information about the attention span and the time needed to process the stimulus/object (Hüttermann et al., 2018). It has been suggested that expert athletes exhibit a distinct gaze behavior, leading to an optimization in the visual information collected and maximizing the coupling between perception and action (Klostermann and Moeinirad, 2020). Indeed, in the reviews of Mann et al. (2007) and Gegenfurtner et al. (2011), it was found that expert athletes presented fewer fixations with longer durations, showing that they focus on different, but more task-relevant information sources. However, in a recent review in the field (Klostermann and Moeinirad, 2020), these findings were not corroborated. A similar pattern among different skill levels was found for both the number and the duration of the fixations. In that same review, a comparison between expert and intermediate skill athletes was performed (*n* = 41 studies), as well as between experts and novices (*n* = 32 studies). In addition, it was also found that the same number of studies noticed a significant negative result in the number of fixations when comparing experts with intermediate and novice athletes (Klostermann and Moeinirad, 2020).

These confusing outcomes were also expressed in the present meta-analysis, shown in Figure 6. In fact, in the sample of 218 evaluated athletes, a small effect (non-significant: ES = 0.39; *p* = 0.280) was observed, not corroborating the older studies in the field. This could be the result of a great heterogeneity found (*I*^2^ = 84.4%) in the present analysis. It is possible that sports scenarios do not always require the same search strategies, to cover the average data. Although this finding does not inhibit experts from using the best strategy for each specific scenario, it may imply a greater number of fixations in one instead of another scenario. Nevertheless, when exploring the number of fixations in each sport, moderate effects were found (although not significant; *p* = 0.078 between groups). However, two main concerns should be highlighted: (i) only three studies in volleyball and four studies in soccer were included, and (ii) the results were quite different for each sport, as shown in Figure 7.

Regarding the fixation duration, in the study of Klostermann et al. (2018) and Moeinirad et al. (2020), a similar number of studies were found to have noticed significant and non-significant results. However, in the present study, more studies were registered, revealing nonsignificant (e.g., Park, 2003; McRobert et al., 2011; Castro et al., 2017; Natsuhara et al., 2020) than significant results (Williams et al., 1994; Williams and Davids, 1997, 1998; Roca et al., 2011), with a small effect observed when analyzing all studies together (Figure 8) and no effect when separated by sport (Figure 9). Once again, this could be due to the heterogeneity observed in the included study (*I*^2^ = 75.1%), showing that there is a lot of dispersion in the results.

**Figure 10:**
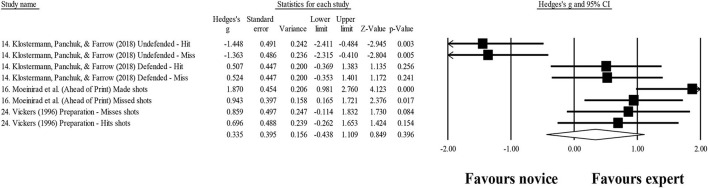
Quiet eye duration: similar quiet eye duration was noted for expert athletes compared to novice athletes. Black squares: individual studies. Its size represents their relative weights. White rhomboid: summary value.

Longer duration on quiet eye has been reported when comparing experts to intermediate and novice athletes (Mann et al., 2007; Gegenfurtner et al., 2011; Klostermann and Moeinirad, 2020). The capacity to predict performance has been associated with expertise (Lebeau et al., 2016), since it was suggested that during this period, task-relevant environmental cues are processed and motor programs are retrieved and coordinated for task success (Vickers, 1996a,b). These findings seem to be in line with those exhibited in Figure 10, although only a small effect was proven to exist (ES = 0.34; *p* = 0.396), instead of a moderate and large effect considered in previous studies (e.g., Lebeau et al., 2016). Nevertheless, in the present study, although experts showed a trend to spend more time in the quiet eye, that value was not significant. It should be noted that this variable also showed high heterogeneity values (*I*^2^ = 82.9%). In addition, in line with the Klostermann and Moeinirad (2020) review, in contrast with other gaze measures, no study with significant negative results was found, but only a trend for a higher quiet eye duration in an undefended basketball game situation was reported (Klostermann et al., 2018).

It seems that over the last years, the results reported in this field have changed. In fact, the number of studies that revealed nonsignificant results has overwhelmed the number of studies with significant positive results (Klostermann and Moeinirad, 2020). To explain this finding, researchers have suggested that the main reason for these different results can be attributed to the advances in technology, specifically more accurate and reliable gaze data (Kredel et al., 2017; Orquin and Holmqvist, 2018). Moreover, recent developments in eye trackers allowed to conduct studies in a more ecological and thus more representative environment (Orquin and Holmqvist, 2018), which has been found to affect gaze behavior (e.g., Dicks et al., 2010; van Maarseveen et al., 2015). In fact, as we could observe in Table 2, different eye-tracker brands and specifications were used in different studies. Nevertheless, in future studies, more attention must be paid to the athletes' classification, to have a normalization of the data and the certainty that we are evaluating experts and novices.

It should be noted that the major limitation inherent to the present study was the method used to classify and characterize experts and novices. However, this difficulty and diversity of criteria have already been reported and discussed elsewhere (Chi, 2006; Swann et al., 2015; McKay et al., 2022), which may confuse the results and conclusions drawn.

## Data availability statement

The original contributions presented in the study are included in the article/supplementary material, further inquiries can be directed to the corresponding author/s.

## Author contributions

AFS, JA, and EM-C: conceptualization and supervision. AFS, JA, AS, NP, and FG: writing. RL, HC, IT, and HS: search and paper screening. RR-C: statistical analysis. AS and JA: project administration. AK and AO: writing revision. All authors have read and agreed to the published version of the manuscript.

## Conflict of interest

The authors declare that the research was conducted in the absence of any commercial or financial relationships that could be construed as a potential conflict of interest.

## Publisher's note

All claims expressed in this article are solely those of the authors and do not necessarily represent those of their affiliated organizations, or those of the publisher, the editors and the reviewers. Any product that may be evaluated in this article, or claim that may be made by its manufacturer, is not guaranteed or endorsed by the publisher.
